# Photo-Responsive Polymersomes as Drug Delivery System for Potential Medical Applications

**DOI:** 10.3390/molecules25215147

**Published:** 2020-11-05

**Authors:** Wanting Hou, Ruiqi Liu, Siwei Bi, Qian He, Haibo Wang, Jun Gu

**Affiliations:** 1 Department of Medical Oncology Cancer Center, West China Hospital, Sichuan University, Chengdu 610000, Sichuan, China; hwt9229@126.com; 2 College of Biomass Science and Engineering, Sichuan University, Chengdu 610000, Sichuan, China; 3 Department of Burn and Plastic Surgery, West China Hospital, Sichuan University, Chengdu 610000, Sichuan, China; rachael91liu@163.com (R.L.); bisiwei@yeah.net (S.B.); 4 Department of Emergency, West China Hospital, Sichuan University, Chengdu 610000, Sichuan, China; raynaud2012@163.com; 5 Department of Cardiovascular Surgery, West China Hospital, Sichuan University, Chengdu 610000, Sichuan, China

**Keywords:** photo-responsive polymersomes, hydrophilic drugs, hydrophobic drugs

## Abstract

Due to a strong retardation effect of o-nitrobenzyl ester on polymerization, it is still a great challenge to prepare amphiphilic block copolymers for polymersomes with a o-nitrobenzyl ester-based hydrophobic block. Herein, we present one such solution to prepare amphiphilic block copolymers with pure poly (o-nitrobenzyl acrylate) (PNBA) as the hydrophobic block and poly (*N,N’*-dimethylacrylamide) (PDMA) as the hydrophilic block using bulk reversible addition-fragmentation chain transfer (RAFT) polymerization of o-nitrobenzyl acrylate using a PDMA macro-RAFT agent. The developed amphiphilic block copolymers have a suitable hydrophobic/hydrophilic ratio and can self-assemble into photoresponsive polymersomes for co-loading hydrophobic and hydrophilic cargos into hydrophobic membranes and aqueous compartments of the polymersomes. The polymersomes demonstrate a clear photo-responsive characteristic. Exposure to light irradiation at 365 nm can trigger a photocleavage reaction of o-nitrobenzyl groups, which results in dissociation of the polymersomes with simultaneous co-release of hydrophilic and hydrophobic cargoes on demand. Therefore, these polymersomes have great potential as a smart drug delivery nanocarrier for controllable loading and releasing of hydrophilic and hydrophobic drug molecules. Moreover, taking advantage of the conditional releasing of hydrophilic and hydrophobic drugs, the drug delivery system has potential use in medical applications such as cancer therapy.

## 1. Introduction

Photo-responsive polymersomes (i.e., polymer vesicles) have attracted great interest as controlled drug delivery systems since they can realize spatial and temporal control of the release of the encapsulated therapeutics on demand via light irradiation [1,2,3]. The key to constructing these polymersomes is to confer amphiphilic block copolymers with photo-responsiveness by introducing photosensitive moieties called chromophores into polymer chains. Depending on the types of chromophores adopted, two different photo-responsive polymersomes have been developed. As light is applied, they are able to respond in a reversible or irreversible way [4]. The reversible photosensitive moieties include azobenzene [5,6,7], spiropyran [8,9], dithienylethene [10], and diazonaphthoquinone [11]; while the irreversible ones include o-nitrobenzyl [12,13,14,15,16,17,18,19], pyrenylmethyl [14], and coumarin [20,21,22].

Among these photosensitive moieties, o-nitrobenzyl and its derivatives have gained intensive attention for the applications of controllable drug delivery [23,24,25]. o-nitrosobenzaldehyde is a commonly used molecule in designing drug delivery systems for cancer therapy and nitrosobenzaldehyde shows a low toxicity to the body [26,27]. Under light irradiation, they perform irreversible photocleavage isomerization and generate a corresponding o-nitrosobenzaldehyde and a free carboxylic acid. Along these lines, some properties of the photoresponsive polymersomes will substantially alter. The o-nitrobenzyl group, for example, has been inserted at the block junction between the hydrophobic and hydrophilic blocks of the amphiphilic block copolymers. Upon light irradiation, photocleavage of the o-nitrobenzyl groups disturb the bilayer membrane of the corresponding polymersomes, which re-organize into smaller polymersomes accompanied by the release of the loaded drugs. This strategy has been adopted by Katz and Meier to deliver different payloads including biocytin, drugs, and biomolecules [28,29]. However, this strategy is more favorable for releasing the hydrophilic drugs. Another strategy to construct photoresponsive polymersomes based on o-nitrobenzyl ester is introducing o-nitrobenzyl groups to the side chains of the hydrophobic block. However, due to the strong retardation effects of o-nitrobenzyl groups on polymerization, it is still a great challenge to prepare amphiphilic block copolymers for self-assembling into polymersomes with o-nitrobenzyl ester-based hydrophobic blocks by controlled radical polymerization. Special efforts have been dedicated to the design and synthesis of polymerizable monomers with o-nitrobenzyl far from (meth)acrylate groups [30]. He and coworkers have designed a novel photolabile monomer with o- nitrosobenzaldehyde acetal and prepared photoresponsive polymersomes from this photolabile acetal containing amphiphilic block copolymers. The developed polymersomes can realize the controlled release of hydrophilic cargo via light and pH stimuli [31]. Liu and coworkers reported amphiphilic block copolymers containing photolabile carbamate-caged primary amines with ethylene separation from o-nitrobenzyl groups. The resultant polymersomes demonstrate a photo-induced traceless crosslinking behavior that can realize polymersome stabilization and tune bilayer membrane permeabilization on demand [20]. Therefore, it has been successfully applied to achieve the co-release of both hydrophobic and hydrophilic substances. Despite the great promise of these polymersomes, it is still innovative to develop a simple and general strategy to prepare amphiphilic block copolymers for self-assembling into polymersomes using o-nitrobenzyl acrylate as the starting materials via a routinely-adopted controlled polymerization method.

**Scheme 1:**
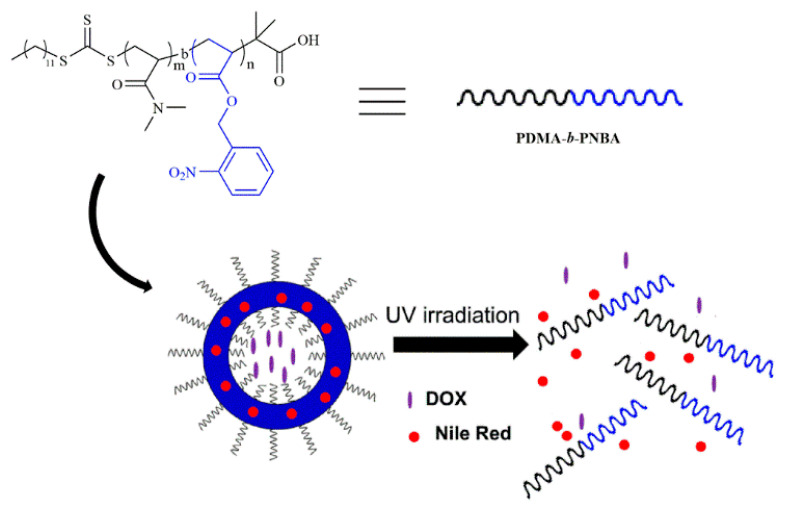
Schematics for the controllable light-responsive co-release of hydrophobic and hydrophilic drugs from the developed polymersomes of PDMA*-b-*PNBA. DOX: doxorubicin; PDMA*-b-*PNBA: *N,N’*-dimethylacrylamide-*b*-o-nitrobenzyl acrylate; UV: ultraviolet radiation.

Herein, we present one solution to prepare an amphiphilic block copolymer with pure poly (o-nitrobenzyl acrylate) (PNBA) as the hydrophobic block and poly (*N,N’*-dimethylacrylamide) (PDMA) as the hydrophilic block by bulk RAFT polymerization of o-nitrobenzyl acrylate using a PDMA macro-RAFT agent (PDMA–CTA). Shown in Scheme 1, photoresponsive polymersomes can be fabricated from self-assembling of the developed amphiphilic block copolymers and used to co-load hydrophobic and hydrophilic cargos into hydrophobic membranes and aqueous compartments of the polymersomes. Upon UV light irradiation, the hydrophobic block is transformed into an hydrophilic one in the photocleavage reaction of o-nitrobenzyl groups, thereby resulting in dissociation of the polymersomes with simultaneous co-release of both hydrophilic and hydrophobic cargoes. While the UV light could limit its application to a certain extent, many kinds of drug delivery systems respond to the UV irradiation, which has been prepared and studied in lots of labs [26,32,33]. We have reason to believe this unique on-demand releasing characteristic provides a noninvasive drug releasing strategy and could be applied in many clinical areas such as medical imaging and targeted therapy.

## 2. Results

### 2.1. Synthesis of Photoresponsive Block Copolymers 

**Scheme 2:**
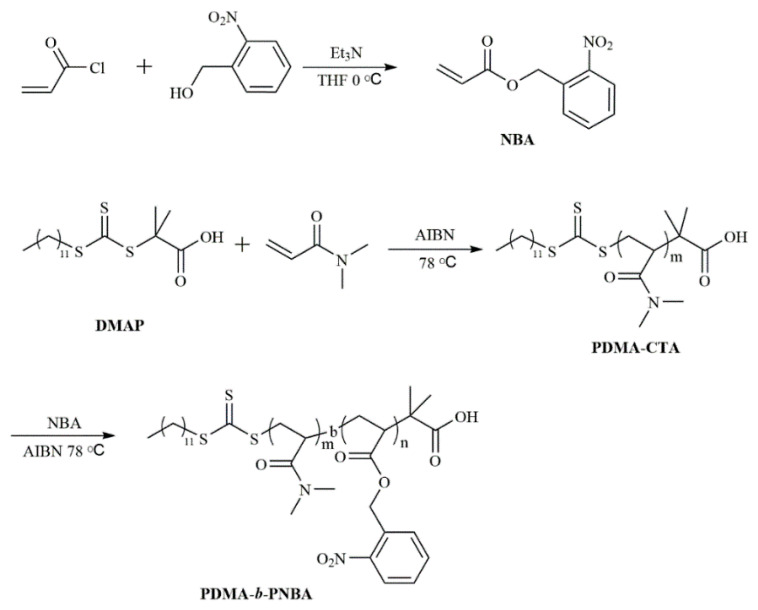
Detailed synthetic route to PDMA*-b-*PNBA.

**Figure 1:**
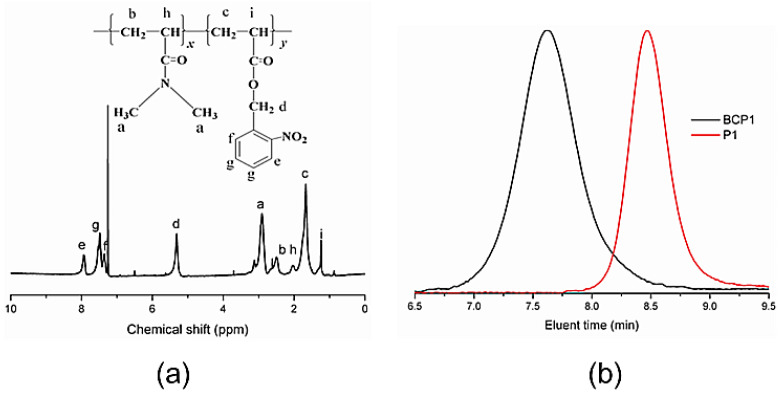
Characterization of PDMA_30_*-b-*PNBA_97_: (**a**) its ^1^H-NMR spectrum in CCl_3_D; and (**b**) the GPC elution curve in THF using polystyrene standards for calibration. CCl_3_D: chloroform-d; THF: tetrahydrofuran.

To fabricate a photosensitive polymer, a photoresponsive monomer NBA was synthesized according to the literature [28]. Then, the photoresponsive amphiphilic block copolymers were synthesized via RAFT polymerization. The detailed synthetic route is shown in Scheme 2. The PDMA polymer with a polymerization degree of 30 (PDMA–CTA) was first synthesized by RAFT polymerization and selected as the hydrophilic block. Subsequently, the diblock copolymers were fabricated by RAFT bulk polymerization of o-nitrobenzyl acrylate using a PDMA macro-RAFT agent at 80 ℃. Depending on the reaction time, the block copolymers with different block lengths of poly (2-Nitrobenzyl Acrylate) (PNBA) can be prepared with different conversions of NBA monomers. One thing to note is that a maximum conversion of 60 mol% of (2-Nitrobenzyl Acrylate) (NBA) can be achieved by our method, which endows us with the ability to prepare suitable amphiphilic block copolymers for polymersome self-assembly. The successful synthesis of the diblock copolymers was proved by ^1^H-NMR. Using the ^1^H NMR spectrum, the composition of (*N,N’*-dimethylacrylamide_30_-b-o-nitrobenzyl acrylate_97_) PDMA_30_*-b-*PNBA_97_ is determined in Figure 1a by comparing the integrals of the resonance peaks of a methene proton of o-nitrobenzyl at 5.2 ppm and methyl groups of PDMA at 2.9–3.2 ppm. Furthermore, the gel permeation chromatography (GPC) of PDMA_30_*-b-*PNBA_97_ that can self-assemble into polymersomes is shown in Figure 1b. The theoretical molar mass of this polymersome is about 23,049 g/mol. The GPC trace of the target diblock copolymer clearly shifts to the higher molecular weight when compared to that of the PDMA block but maintains its low molecular dispersity. It reveals that the PDMA_30_*-b-*PNBA_97_ have a low molecular dispersity index of 1.25 with an average number molecular weight of 20.5 kg/mol.

### 2.2. Photoresponsive Block Copolymers Photocleavage 

**Figure 2:**
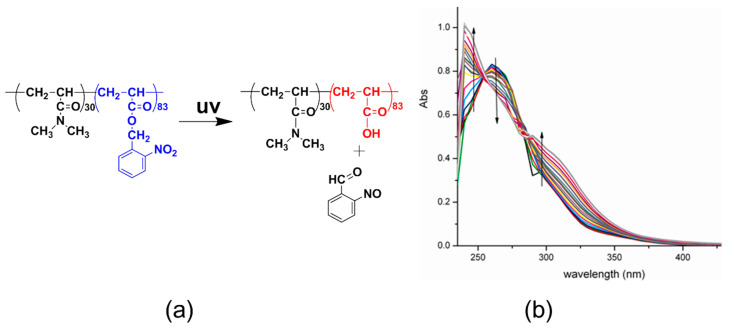
Photocleavage reaction of PDMA_30_*-b-*PNBA_97_ upon exposure to light irradiation at 365 nm. (**a**) Schematic of the photocleavage of the polymer showing the cleavage product and the shift of hydrophobic and hydrophilic balance; (**b**) UV-vis spectra of the polymer solution in THF with different irradiation times. PDMA_30_*-b-*PNBA_97_: *N,N’*-dimethylacrylamide_30_-b-o-nitrobenzyl acrylate_97_.

To ensure that the developed polymersomes possess the photo-triggered disassembly for controlled drug release, the photo-responsive property of the PDMA_30_*-b-*PNBA_97_ was first monitored by UV-vis spectroscopy. Shown in Figure 2, light irradiation of its polymer solution at 365 nm induces dramatic evolution of the UV-vis absorption spectra. Specifically, the absorbance at 245 and 325 nm gradually increased while the absorbance at 350 nm decreased; two isosbestic points generated 289 nm, and 263 nm, respectively. These results indicate that an o-nitrobenzyl ester of the hydrophobic block carries out an efficient and clean photocleavage reaction to generate an o-nitrosobenzaldehyde and a free carboxylic acid, as shown in Figure 1a. Furthermore, the amphiphilic block copolymers PDMA_30_*-b-*PNBA_97_ would transfer into a double hydrophilic block copolymer PDMA*-b-*PAA, as shown in Figure 1b, providing the premise for triggering dissociation of polymersomes.

**Figure 3:**
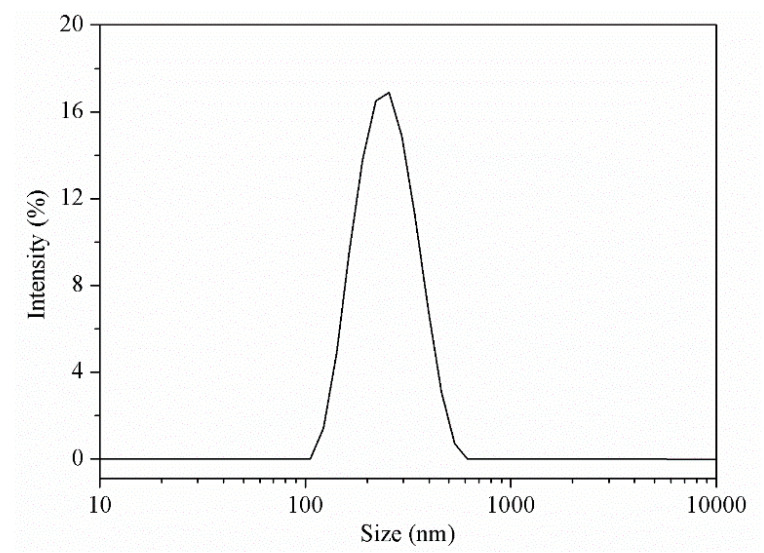
DLS plots of polymersomes.

**Figure 4:**
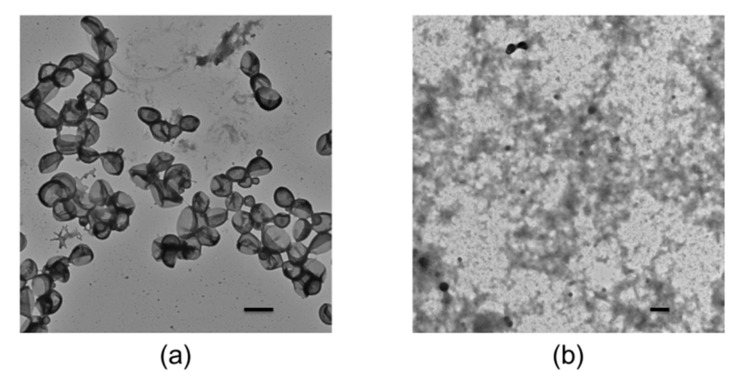
TEM characterizations of polymersomes (**a**) before and (**b**) after UV irradiation. The scale bars in the insets are 200 nm.

The polymersomes were prepared by self-assembly of the PDMA_30_*-b-*PNBA_97_ copolymers in water/chloroform emulsions followed with complete removal of chloroform via gradual evaporation. The size of the polymersomes also was measured by dynamic light scattering (DLS) using a Zetasizer Nano-ZS from Malvern Instruments equipped with an He-Ne laser at a wavelength of 633 nm at 25 °C and the measurement of sucrose at a 173° detection angle. The results indicated the diameter of the polymersomes was about 200 ± 30 nm (Figure 3). Furthermore, transmission electron microscope (TEM) characterization revealed that the formed polymersomes had an average diameter of about 150 nm with a membrane thickness of about 30 nm (Figure 4a). The size measured by TEM was smaller than that of DLS. The possible reason was the shrinkage of the shell upon drying. Shown in Scheme 1, the vesicular microstructure should be composed of hydrophobic PNBA like the bilayer membrane and hydrophilic PDMA as inner and outer coronas. Therefore, upon light irradiation, the cleavage of o-nitrobenzyl ester groups would increase the polarity of the hydrophobic block, thereby disturbing the hydrophobic-to-hydrophilic balance. Once enough cleavage of the o-nitrobenzyl ester groups occurs, the polymersomes will be disrupted or fall apart because the block copolymers are hydrophilic enough to be dissolved in an aqueous solution. This was confirmed by TEM characterization of the UV-irradiated polymersome dispersion. Shown in Figure 4b, after 15 min of light irradiation at 365 nm, the vesicular microstructures have totally disappeared, and only irregular small aggregates can be observed under TEM. All these results confirm that the developed polymersomes can realize photo-controlled disassembly of a PNBA-based bilayer membrane with simultaneous release of aqueous compartments, providing the great potential for loading and co-releasing of hydrophobic and hydrophilic cargos. 

### 2.3. Photo-Controlled Co-Release of Hydrophobic and Hydrophilic Drugs

**Figure 5:**
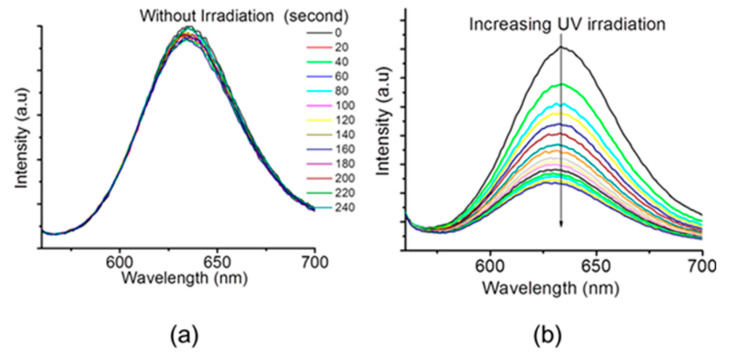
The Polymersomes as carriers for light-controlled release of hydrophobic drugs, NR: (**a**) Emission spectra of NR for the polymersomes solution without UV irradiation; (**b**) Emission spectra of NR for the polymersomes solution under UV irradiation at 365 nm.

To illustrate the potential of the developed polymersomes as controlled drug delivery systems, the hydrophobic and hydrophilic model drugs, doxorubicin hydrochloride (DOX) and Nile red dye (NR), were co-loaded into the polymersomes and the photo-triggered co-release of these two types of drugs was evaluated. Figure 5 shows the light-induced NR release profile upon exposure of the polymersomes solutions to 365 nm UV irradiation. Without UV irradiation, there is no change in the fluorescence intensity of the polymersome solution, suggesting that NR molecules are stably dispersed in the bilayer (Figure 4a), whereas, UV irradiation of the polymersome solution brings in a clear decrease in the fluorescence intensity of NR with increasing irradiation time (Figure 5b). These results confirm that the photo-induced vesicular disassembly can realize successful controlling releases of hydrophobic NR molecules into the aqueous solution as the fluorescence of NR has been quenched.

**Figure 6:**
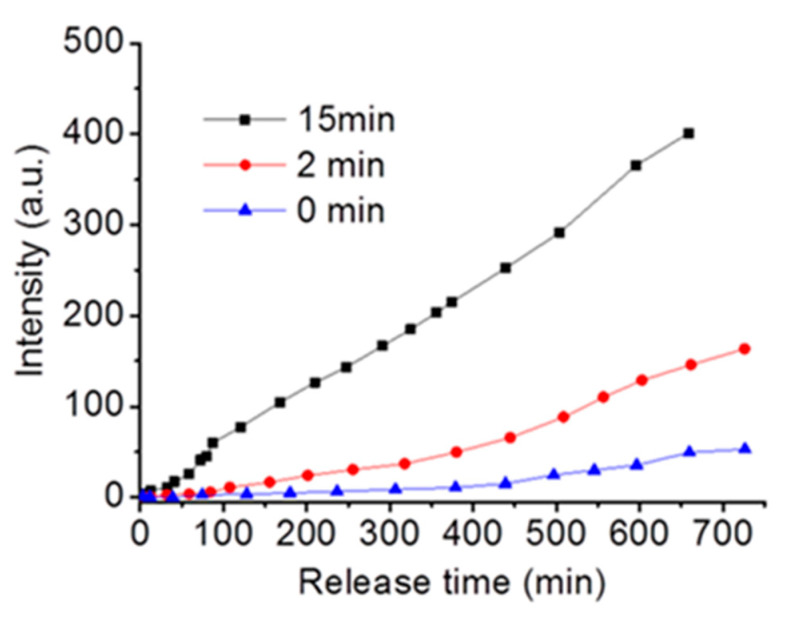
The Polymersomes as carriers for controlled release of hydrophilic drugs, DOX, on exposure to UV light with different times.

Figure 6 demonstrates a light-controlled release of trapped hydrophilic DOX from an aqueous compartment of polymersomes. The increase in the fluorescent intensity of DOX can keep track of the release DOX at the emission maximum wavelength. Without light irradiation, only a slight amount of the DOX molecules can be released from the polymersomes, which is consistent with some previous research [34,35,36]. However, exposing the polymersome solution to UV light at 365 nm for 2 min results in a significant increase in the amount of DOX released from the polymersomes, clearly displaying the light-controlled release of the hydrophilic drug as a result of polymersome dissociation. The release of DOX from the polymersomes is highly dependent on the irradiation time. It expressed four times enhancement in the release rate when the irradiation time was increased to 15 min.

## 3. Materials and Methods 

### 3.1. Materials 

2-Nitrobenzyl alcohol and acryloyl chloride was purchased from Shanghai Haiqu Chemical Co., Ltd. Nile red (Shanghai, China) and 2, 2′-azobisisobutyronitrile (AIBN) was purchased from Aldrich (St. Louis, MO, USA). Dichloromethane (DCM), chloroform, ethyl ether and tetrahydrofuran (THF) were purchased from Aladdin (Shanghai, China). Water was deionized with a Milli-Q SP reagent water system (Millipore (Suzhou, China)) for a specific resistivity of 18.4 MΩ.cm. 2-(Dodecylthiocarbonothioylthio)-2-methylpropionic acid (DMAP) was prepared according to a reported procedure [37]. All the other reagents were purchased from Aladdin Chemical Reagent Co., Ltd. (Shanghai, China) and used as obtained. 

### 3.2. Characterizations

^1^H-NMR spectra were obtained using Deuterated Chloroform as a solvent and Tetramethylsilane as an internal norm on a Bruker 300 MHz spectrometer (Chengdu, China). The spectra were employed to determine the polymerization unit of the block copolymers. Measurements of Gel permeation chromatography (GPC) were carried out on a Waters device fitted with a photodiode array detector (PDA 996) and a refractive index detector (RI 410) (Chengdu, China). GPC setup was comprised of PL gel 10 mm MIXED-B columns using a single refractive index (RI) detector (Chengdu, China) and a series of narrow polystyrene as standards for the calibration curve. The eluent was THF, and the flow rate was 1.0 mL·min^−1^ at 40 °C. THF was used as the eluent at an elution rate of 1 mL/min, while polystyrene standards were used for calibration. Photoreaction of the block copolymer as a function of UV irradiation time was recorded on a Varian 50 Bio UV-vis spectrophotometer (Chengdu, China). The fluorescence emission spectra were recorded on a fluorescence spectrophotometer (Varian Cary Eclipse) (Chengdu, China). The morphologies of the polymer vesicles were inspected using a Hitachi H-7500 transmittance electron microscope (TEM) (Chengdu, China) operating at 60 kV. Regarding TEM measurement, a drop of the solution was spread on a TEM copper grid. The solution remained for 5 min and the excess solution was blotted off using filter paper and allowed to dry at room temperature overnight. 

### 3.3. Synthesis Procedures

Synthesis of 2-Nitrobenzyl Acrylate (NBA) Monomer. The monomer was synthesized according to a reported procedure with some modifications [38]. Typically, 2-Nitrobenyl alcohol (1.5 g, 9.8 mmol, 1.0 equiv.) and triethylamine (1.09 g, 10.78 mmol, 1.1 equiv.) were dissolved in THF (30 mL) at 0 ℃ to form a homogeneous solution. After cooling for 30 min, a solution of acryloyl chloride (0.745 g, 10.78 mmol, 1.1 equiv.) dissolved in 20 mL THF was added dropwise into the mixture under magnetic stirring. The whole mixture was stirred at 0 ℃ for 2 h and then at room temperature overnight. After removing all the solvent under reduced pressure, the crude product was dissolved in DCM, washed with HCl (0.1 M) and saturated brine, then dried over MgSO_4_. The product was collected by filtration and then purified by flash column chromatography on silica gel with DCM as an eluent, which was obtained as a light-yellow oil (1.3 g).

RAFT Synthesis of PDMA macro-RAFT agent. RAFT polymerization of DMA was conducted at 70 ℃, employing AIBN as the radical initiator and DMAP as the RAFT chain transfer agent. A typical example is as follows: DMA (3 g, 0.03 mol), DMAP (0.365 g, 0.001 mol), AIBN (0.0324 g, 0.0002 mol) were added to a 25 mL one-pot round flask. Then, 3 mL of anhydrous anisole was included to dissolve the blend. After purging 30 min with argon of high purity, the whole system was immersed into a preheated oil bath for 4 h at 78 ℃ with constant stirring. The crude product was purified by precipitating twice into excess ethyl ether, followed by vacuum-drying. The monomer conversion was approximately 100% as measured from the ^1^H-NMR spectrum. The sample was obtained as a PDMA_30_ macroRAFT agent (PDMA-CTA).

RAFT Synthesis of PDMA*-b-*PNBA Diblock Copolymer. PDMA-CTA was used as the chain transfer agent to prepare the block copolymer. PDMA-CTA (0.075 g, 0.025 mmol), AIBN (0.0016 g, 0.001 mmol) were dissolved in NBA (1.3 g, 5.28 mmol). After purging Argon for 30 min, the reaction was continued for 24 h at 80 ℃ with constant stirring. The product was purified by precipitating three times into excess cold hexane, followed by vacuum-drying overnight. The sample was obtained as a yellowish powder (0.65g, 50% yield). GPC PDMA_30_*-b-*PNBA_97_.

Vesicle Preparation and Drug Loading/Photo-Controlled Release. Regarding self-assembly of the block copolymer in water, water was added to a solution of the polymer (5 mg per mL) in chloroform and the two-phase mixture was stirred vigorously for three days. After evaporation of the organic solvent, the aqueous phase was dialyzed against deionized water for 24 h. The final concentration of the block copolymer was 2.5 mg per mL.

Doxorubicin Encapsulation. An aqueous solution of Doxorubicin hydrochloride (DOX) was added to a solution of the block copolymer (5 mg per mL) in chloroform and the two-phase mixture was stirred vigorously for three days. After evaporation of the organic solvent, the aqueous phase was dialyzed against deionized water for 24 h. Non-entrapped DOX was removed by dialysis against water for 3 days. The final concentration of the polymer was 2.0 mg per mL.

Nile red Encapsulation. Twenty-four microliters of a 0.5 mg per mL stock solution of Nile red in dichloromethane was added to an empty vial, which was then placed under vacuum for at least two hours to ensure complete solvent removal. To this vial was added 1 mL of 2.5 mg per mL aqueous vesicular solution. This solution was stirred vigorously at room temperature for at least 4 h, and then filtered with a column of cotton.

Drug loading efficiency (DLE) was calculated according to the following Equation (1):

DLE (%) = (weight of loaded drug/weight in feed) × 100%(1)

The DLE of DOX and Nile red were determined to be 81% and 75%, respectively.

Photo-Controlled Release of a Hydrophilic Drug upon UV Irradiation. The drug release experiments were carried out as follows: A UV cell filled with 3 mL pure water was carefully closed with the dialysis cap whose membrane was immersed in water. Then, 0.3 mL of DOX-loaded vesicle solution, after UV irradiation or no irradiation, was poured into the dialysis cap. Sampling the solution underneath the dialysis membrane enabled the measurement of the fluorescence emission of DOX that is released from the vesicle solution and diffuses into the cell through the membrane. The release kinetics of DOX was recorded following irradiation for certain time periods.

Photocontrolled Release of a Hydrophobic Drug upon UV Irradiation. The stock NR-loaded vesicular solution was a diluted system, and the fluorescence measurements were taken. The above procedure was repeated.

## 4. Conclusions

To summarize, we developed a simple and general strategy to prepare amphiphilic block copolymers using o-nitrobenzyl acrylate as the starting material via a routinely-adopted controlled polymerization method. This method achieved a maximum conversion of 60 mol% of NBA in our polymerization conditions, endowing it with the ability to prepare an amphiphilic block copolymer, PDMA_30_*-b-*PNBA_97,_ with a suitable hydrophobic/hydrophilic ratio for self-assembly of polymersomes. The resultant polymersomes realized controlled dissociation upon light irradiation and were used to load and release hydrophobic and hydrophilic drugs at the same time in a controlled means by tuning light irradiation time. We expect that the reported strategy can greatly simplify the synthesis of other o-nitrobenzyl-based block copolymers for developing photoresponsive polymersomes based on o-nitrobenzyl as a smart drug delivery nanocarrier for controllable co-release of hydrophilic and hydrophobic drug molecules.
